# Stabilizing the West Antarctic Ice Sheet by surface mass deposition

**DOI:** 10.1126/sciadv.aaw4132

**Published:** 2019-07-17

**Authors:** Johannes Feldmann, Anders Levermann, Matthias Mengel

**Affiliations:** 1 Potsdam Institute for Climate Impact Research, Potsdam, Germany.; 2 Lamont-Doherty Earth Observatory, Columbia University, Palisades, NY, USA.; 3 Institute of Physics, Potsdam University, Potsdam, Germany.

## Abstract

There is evidence that a self-sustaining ice discharge from the West Antarctic Ice Sheet (WAIS) has started, potentially leading to its disintegration. The associated sea level rise of more than 3m would pose a serious challenge to highly populated areas including metropolises such as Calcutta, Shanghai, New York City, and Tokyo. Here, we show that the WAIS may be stabilized through mass deposition in coastal regions around Pine Island and Thwaites glaciers. In our numerical simulations, a minimum of 7400 Gt of additional snowfall stabilizes the flow if applied over a short period of 10 years onto the region (−2 mm year^−1^ sea level equivalent). Mass deposition at a lower rate increases the intervention time and the required total amount of snow. We find that the precise conditions of such an operation are crucial, and potential benefits need to be weighed against environmental hazards, future risks, and enormous technical challenges.

## Teaser

Collapse of the West Antarctic Ice Sheet can in principle be stopped by artificial snowfall but challenges are large and manifold.

## INTRODUCTION

Dynamic ice loss from Pine Island Glacier (PIG) and Thwaites Glacier (TG) currently represents Antarctica’s largest and strongly accelerating contribution to global sea level rise. From the mid-1990s to the year 2010, the ice loss from both regions substantially increased from 6 Gt year^−1^ to more than 40 Gt year^−1^ for PIG and from about 30 to 52 Gt year^−1^ for TG (*1*). Recent estimates from the IMBIE (Ice Sheet Mass Balance Inter-comparison Exercise) project show an increase of West Antarctica’s sea level contribution from 0.15 mm year^−1^ in 1992 to 0.44 mm year^−1^ in 2017 (*2*). This acceleration in ice discharge is likely linked to the increased flow of warmer ocean waters toward the ice shelves (*3*–*5*). The warm water masses melt the ice shelves adjacent to the glaciers from below. Thinner ice shelves exert less backstress (buttressing) on their feeding glaciers (*6*, *7*), increasing glacier flow speed. The consequent thinning of the glaciers presumably triggered the grounding line retreat of several tens of kilometers inland (*8*, *9*). Both PIG and TG rest on inland-sloping bedrock below sea level, which makes them prone to the marine ice sheet instability, i.e., a self-sustaining discharge due to the increase in ice thickness at the grounding line during the retreat (*10*–*14*). Numerical simulations support the hypothesis that an early stage of collapse has already begun at both sites (*15*–*19*) and may lead to a 40-km unstable retreat of the PIG grounding line over the coming 20 years with an accelerated ice loss of 100 Gt year^−1^ or even more (*16*). In two of three simulations with different regional ice sheet models, the retreat is irreversible unless the basal melt rates are strongly reduced compared to present-day control conditions. Even after the grounding line reaches the bottom of the marine basin, the simulated rates of ice loss from PIG remain elevated at 60 to 120 Gt year^−1^. Numerical simulations of TG show an increased discharge in the later phase of retreat in about 200 to 900 years (*15*). This future retreat releases 361 Gt year^−1^ of ice to the ocean, corresponding to a sea level rise of 1 mm year^−1^ under a wide range of different assumptions regarding basal melt rates including present-day control conditions. The potential long-term sea level rise due to the instability of the marine ice sheet is estimated to be 1.2 m from the Amundsen Sea sector or 3.3 m if the entire marine part of West Antarctica was affected (*20*). This scenario is independent of the question of whether natural oceanic variability or human activity caused the initiation of the instability (*21*–*23*).

Most climate models indicate an increase in snowfall over Antarctica under future global warming (*24*), although the underlying changes in the dynamics of the wind fields and ocean currents in the Southern Ocean need further studies (*25*, *26*). This presumed increase in snowfall is however found to be insufficient to stop the decay of PIG (*16*). The sensitivity to increased snow accumulation was also investigated for TG (*15*). A 20% linear increase in accumulation may stabilize the retreat of TG under low basal melt rates but can only delay the onset of the rapid collapse of the ice sheet under higher melt rates. The long-term evolution of the potential disintegration of the West Antarctic Ice Sheet (WAIS) may be affected by several further processes. For instance, isostatic rebound of the glacial bed during ice sheet retreat is suggested to delay or limit its retreat (*27*–*29*). In contrast, hydrofracturing of ice shelves (*30*) and a corresponding reduction in ice shelf buttressing might amplify an ongoing instability. In addition, the currently debated marine ice cliff instability (*31*, *32*) could accelerate ice sheet decay. Whether the instability was caused by human interference or is part of longer-term natural cycle (*21*, *22*) requires further studies. A number of different techniques have been suggested to stall the self-amplifying discharge from the WAIS (*33*, *34*).

Here, we investigate the option to stabilize both glaciers by pumping ocean water onto the critical area conducting regional simulations with the three-dimensional Parallel Ice Sheet Model (PISM). The model applies a superposition of the shallow ice (*35*) and the shallow shelf approximation (*36*, *37*) of the stress balance (*38*) that ensures a smooth transition between the different flow regimes of an ice sheet, ranging from bed-frozen inland ice over well-lubricated, fast-flowing ice streams to free-floating ice shelves (see Materials and Methods). Stress transmission across the grounding line allows for the effect of ice shelf buttressing (*6*, *7*). PISM’s grounding line is free to evolve, and reversible grounding line motion that is comparable to results from full-Stokes simulations was demonstrated in the MISMIP3d benchmark for horizontal resolutions of 5 km and finer (*39*, *40*).

## RESULTS

**Fig. 1:**
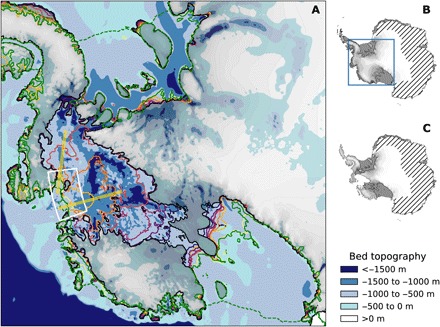
Destabilization of the WAIS in the unperturbed reference simulation. (**A**) Grounding line and calving front of present-day observed state (green contours) and evolution of grounding line position during self-sustained retreat (colored contours, 750-year time steps), underlaid by bed topography (blue shading). The mass addition region used in the perturbation simulations is highlighted by the white sector, within which mass deposition is restricted to ice sheet areas that have been grounded at the onset of the perturbation. Insets show the state of the Antarctic Ice Sheet as observed (**B**) and after simulated collapse of the WAIS (**C**). Hatched area refers to the region outside the model domain, and blue rectangle indicates region shown in (A).

**Fig. 2:**
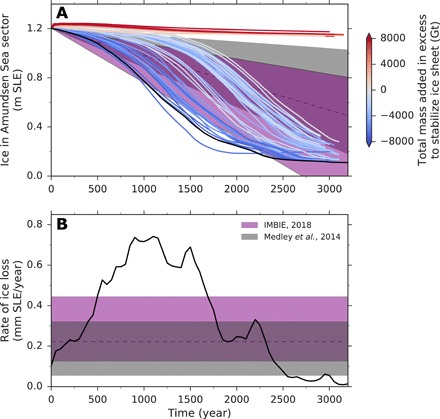
Time series of ice loss from the Amundsen Sea sector for the cases of unperturbed destabilization (black) and mass deposition (colored). (**A**) Changes in sea level relevant ice volume. (**B**) Rate of ice loss. Colors of the curves indicate surplus (red) or lack (blue) in added mass with respect to stabilization threshold, *M*_c_ (see Fig. 4B). Shaded areas indicate the ranges of observed present-day rates of ice loss from the WAIS [purple, 66th percentile with median dashed; (*2*)] and the Amundsen Sea sector [gray; (*1*)]. Continuation of these trends into the future is illustrative, as the drivers of these trends are still disputed. SLE, sea level equivalent.

Our model domain comprises the entire WAIS, the Antarctic Peninsula, and part of East Antarctica (Fig. 1). We start from an ice sheet that was spun up into thermal equilibrium under constant present-day conditions of the atmosphere and the ocean and under fixed bed and ice geometry. Sub–ice shelf melt rates are calculated via the novel Potsdam Ice-Shelf Cavity Model (PICO) module (*41*), which is an integral part of PISM. PICO simulates the vertical overturning circulation in ice shelf cavities, reproducing observed melt rate patterns and the wide range of ice shelf individual average melt rates. Here, we force PICO by circum-Antarctic present-day observations of ocean temperature and salinity (*42*). Prescribed present-day surface mass balance and temperature are taken from the regional atmospheric climate model RACMO (*43*). Letting the ice sheet evolve freely, inland-spreading ice thinning and acceleration, as well as grounding line retreat (Fig. 1), occur with a marine ice sheet instability starting to unfold in the coastal region of TG, which is consistent with present-day observations (*8*, *44*) and recent modeling (*15*–*17*). Initial rates of ice loss are within the range of recent observations (*1*, *2*) and increase with time (Fig. 2). The simulated initial thinning in the coastal area of PIG and TG reaches up to 5 m year^−1^ locally, which is in accordance with observational estimates of maximum thinning rates of 4 to 9 m year^−1^ in that area (*45*, *46*). In this reference simulation, the destabilization of the WAIS takes place unperturbed with ice loss peaking after about 1000 year at sea level contribution rates of almost 1 mm year^−1^. The rates are in line with regional simulations of the destabilization of TG that used a different ice sheet model (*15*). The joint catchment basin of PIG and TG (fig. S1) is drained within 4000 year, confirming results from a previous study applying an earlier version of the PISM model under different boundary conditions (*47*).

We examine the possibility of preventing this destabilization conducting an ensemble of perturbation simulations. We artificially enhance snowfall onto the ice sheet within a fixed sector that covers the coastal region of PIG and TG (Fig. 1). This perturbation is applied as a pulse of mass addition rates between *R* = 62.5 and 875 Gt year^−1^, lasting over a duration of *T* = 10 to 50 year, which yields a total ice mass of *M* = 625 to 18,750 Gt throughout the ensemble of simulations. This would lead to a sea level drop between about 2 and 5 cm, assuming that the mass is taken out of the ocean. We restore the original atmospheric conditions after the perturbation. The oceanic boundary condition remains unmodified during all simulations.

**Fig. 3:**
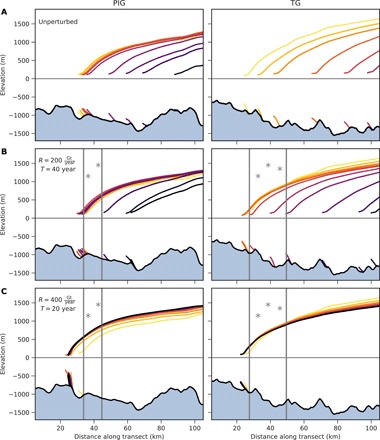
Evolution of PIG and TG profiles in 250 year time steps. (**A**) The unperturbed reference simulation and (**B** and **C**) a mass addition of *M* = 8000 Gt for two different combinations of rate *R* and duration *T* of the perturbation. While the low-rate perturbation (**B**) cannot prevent ice sheet collapse, the high-rate perturbation (**C**) is sufficient to stabilize the ice sheet (see corresponding white stars in Fig. 4). The two vertical lines indicate the sections of mass addition (asterisks) along the transects. The ice shelf is truncated 25 km downstream of the grounding line for clarity. Locations of cross sections are shown in Fig. 1A.

The ice sheet responds with continued and delayed retreat in a part of the simulations, leading to the complete drainage of the marine portion of the WAIS. The other part of simulations reveals a stabilizing ice sheet, which we define as being characterized by grounding line equilibration and a loss in long-term ice volume by less than 5%. Whether stabilization takes place or not depends on whether the amount of added ice is sufficient to stop the previously initiated grounding line retreat. In the unperturbed case, ice sheet thinning, retreat, and discharge evolve in a self-enforcing loop (*10*, *12*). The mass deposition can break this loop through a temporal thickening (~10 m yr^−1^) of the ice sheet in the region adjacent to the grounding line. The enforced increase in ice surface elevation upstream of the grounding line induces a strong steepening of the surface slope, enhancing the driving stress at the grounding line area (*48*). Increased ice advection into the ice shelf (*12*) allows for a regrounding of the ice shelf, pushing the grounding line toward the ocean and up the bed slope. The rate and duration of the perturbation determine whether the local grounding line advance is strong enough to enable the ice sheet to enter a long-term stable configuration (Fig. 3 and figs. S2 and S3).

**Fig. 4:**
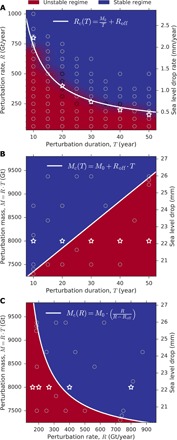
Stability diagrams of the WAIS. (**A**) Rate *R* versus duration *T* of mass addition with unstable regime in red and stable regime in blue. Interpolation of the field is based on the conducted ensemble of stabilization experiments (gray circles). The critical threshold *R*_c_ (white curve) of stabilization is approximated by function given at the top right corner. The approximated threshold is transferred into phase space of (**B**) total amount *M* versus duration of mass addition, and (**C**) total amount versus rate of mass addition. White stars highlight simulations that share the same total amount of deposited mass (*M* = 8000 Gt), added at differing rate and duration, showing that the combination of both determines potential stabilization.

From our ensemble of perturbation experiments, we infer the critical mass addition rate *R*_c_, i.e., the minimum rate to achieve stabilization. We find that it increases with shorter perturbation duration *T* (Fig. 4A): High (low) perturbation rates require a short (long) perturbation duration to affect stability, with minimum rates ranging from about 185 to 735 Gt year^−1^ (sea level equivalent, 0.5 to 2 mm year^−1^). The stabilization threshold curve *R*_c_, which we fit to the data obtained from our simulations, is indirectly proportional to the perturbation duration *T* with an offset *R*_off_. Calculation of the total minimum amount of mass that has to be deposited (*M*_c_ *= R*_c_ · *T*) transforms this threshold to

$$M_{c}(T)=M_{0}+R_{\text{off}}\cdot T$$ (1)

Here, *M*_0_ = 6850 Gt is the theoretical critical value if all ice were added instantaneously. Practically, the mass addition would take place over a period of duration *T*, and Eq. 1 states that, with each year of prolongation of the perturbation period, the stabilization threshold increases by an amount of *R*_off_ = 50 Gt that adds to the theoretical minimum value *M*_0_ (Fig. 4B). Expressing the stabilization threshold *M*_c_ in terms of the mass addition rate yields

$$M_{c}(R)=M_{0}\cdot (\frac{R}{R-R_{\text{off}}})\text{with}\;R>R_{\text{off}}$$ (2)

This critical threshold decreases nonlinearly with increasing mass addition rate *R* (Fig. 4C), asymptotically ranging from *M*_c_(*R* → *R*_off_) = infinity to *M*_c_(*R* → ∞) = *M*_0_. Together, Eqs. 1 and 2 state that, to minimize the total amount of mass to be deposited for stabilization, a strong but short perturbation phase is most favorable. Reducing the perturbation strength requires a prolongation of the perturbation duration, coming at the cost of a higher amount of mass to be added in total. Each year of prolongation increases the total mass to be deposited by about 0.7% with respect to the minimum amount.

## DISCUSSION

Our model simulations neglect several mechanisms that could alter the speed and degree of the potential ice sheet collapse, including possible snowfall increase and ice shelf hydrofracturing due to future atmospheric warming, the effect of glacial isostasy, or consequences of marine ice cliff failure (*24*, *27*–*32*). The absence of these processes introduces uncertainty concerning the ice sheet stabilization threshold inferred in this study. For instance, if ice shelf hydrofracturing, which tends to enhance ice discharge across the grounding line as observed on the Antarctic Peninsula (*49*), would also become relevant for the Amundsen Sea sector, then the amount of mass to be deposited would need to be larger as calculated here. It is also possible that not yet anticipated processes might become important during a potential stabilization effort. Other uncertainties stem from the physical approximations of the ice sheet model, parameter choices, the limited model resolution, observational uncertainties of the used data, and the choice of the mass deposition region in our simulations. Consequently, our study should serve as a proof of concept rather than as a means to deliver exact numbers on the amount and duration of mass deposition required for stabilization. Careful monitoring of the region during the intervention period would need to be a prerequisite to reduce the risk of unintended effects. The practical execution of the endeavor is beyond the scope of this study, and it would be a technical challenge in many ways. For instance, the lifted ocean water could be added either in liquid form or as snow onto the ice sheet. Liquid water addition would raise the question of how it could be kept at the ice sheet surface sufficiently long to freeze. The creation of supraglacial lakes may need to be prevented, as it could cause acceleration of ice discharge as observed in Greenland (*50*). Snowing the water mass onto the ice sheet would mimic the type of precipitation naturally occurring over most part of the ice sheet, demanding a considerable amount of energy and requiring extensive infrastructure for the snow making. The effect of latent heat release due to the phase change of the water is not accounted for in our simulations. In addition, desalination of the water would need consideration, as the addition of salt to the ice sheet surface could have serious effect on the local flow dynamics of the ice sheet. A potential desalination procedure (*51*) would be extremely energy consuming and would need an appropriate, robust, high-end technology. Furthermore, the removal of the sea water to be deposited on the ice sheet would likely perturb the circulation regime of the ocean locally, possibly facilitating the intrusion of warm water into the glaciers’ ice shelf cavities. The additionally induced local flow would be roughly 0.03 sverdrup, which is about 10% of the strength of the ocean circulation beneath the ice shelf cavity of PIG (*52*).

The ocean water would have to be lifted from the sea level to the top of the ice sheet by about 640 m on average (mean ice surface elevation of the perturbation area). In our simulations, this area covers the joint coastal region of PIG and TG with an extent of about 52,000 km^2^, which is similar to the size of the state of Costa Rica or half the size of Iceland. The practical realization of elevating and distributing the ocean water would mean an unprecedented effort for humankind in one of the harshest environments of the planet. For instance, the uplifting of the ocean water alone would require a theoretical minimum of 145 GW (neglecting frictional losses), a power that, in theory, could be provided by more than 12,000 high-end wind turbines driven by the regional wind fields, which in principle would have sufficient capacity (*53*). However, the effective total power demand of the endeavor including potential desalination and heating of the ocean water, as well as snow making, carried out under the difficult circumstances of the Antarctic climate, could be much higher, requiring careful assessment by engineering experts. The building of the wind turbines and the further infrastructure, as well as the extraction of the ocean water itself, would mean the loss of a unique natural reserve, with serious effects on its sensitive marine and coastal ecosystems [e.g., significant emission of underwater noise and electromagnetic fields and fatal collision with energy structure; see (*54*)]. Potential hazards coming along with such an enormous operation would be difficult to anticipate and hard to handle, likely having devastating impact. Despite its disruptive character, the intervention could however relieve the world’s most populous areas from the long-term, several meter–scale sea level commitment of a tipped WAIS. Whether the projected continuation of the observed destabilization of the ice sheet will prove true has to be answered by extensive future monitoring of the Amundsen Sea sector, forming the basis for a weighing between the benefits and the serious implications of an artificial restabilization of the ice sheet. Operations such as the one discussed pose the risk of moral hazard. We therefore stress that these projects are not an alternative to strengthening the efforts of climate mitigation. The ambitious reduction of greenhouse gas emissions is and will be the main lever to mitigate the impacts of sea level rise. The simulations of the current study do not consider a warming ocean and atmosphere as can be expected from the increase in anthropogenic CO_2_. The computed mass deposition scenarios are therefore valid only under a simultaneous drastic reduction of global CO_2_ emissions.

## MATERIALS AND METHODS

### Ice sheet model

We used PISM (*55*, *56*) to carry out regional simulations of the WAIS at a horizontal resolution of 4 km and a minimum vertical resolution of 7 m. The model applies a superposition of the shallow ice approximation (*35*) and the shallow shelf approximation (*36*, *37*) of the full-Stokes stress balance (*38*). This hybrid scheme ensures a smooth transition between different ice sheet flow regimes and allows for stress transmission across the grounding line. A linear interpolation of the freely evolving grounding line and, accordingly, interpolated basal friction enable realistic grounding line motion also at medium or low resolution (*40*). Basal friction was calculated using a nonlinear Weertman-type sliding law (*38*) with a sliding exponent of 3/4 in combination with a Mohr-Coulomb model for plastic till (*57*, *58*) that accounts for the effect of evolving ice thickness and the associated change in overburden pressure on the basal till (*59*). The till friction angle was parameterized with bed elevation, as in (*60*), and the basal pore water pressure was limited to a maximum fraction of 0.97 of the overburden pressure. This friction scheme ensures a continuous transition from quasi–nonslip regimes in elevated regions to the marine areas where basal resistance was low. We used a kinematic first-order calving law (*61*, *62*), with a prescribed proportionality factor of 10^17^ m·s and a minimum ice thickness at the calving front of 200 m. The physically motivated calving law takes into account the eigenvalues of the horizontal strain-rate tensor, allowing for geographically confined ice shelves and dynamic calving front positions. A physical stress boundary condition was applied at the calving front to close the equations of the shallow shelf approximation (*56*).

### Boundary conditions

The landward boundary of our model domain was chosen along present-day East Antarctic ice divides (*63*) and hence far away from the marine parts of the WAIS. Along this fixed boundary, ice velocities were set to zero, whereas the coastal ice margin was free to evolve. The perturbation simulations were initialized from an ice sheet that was spun up for 100,000 model years under present-day constant conditions of the ocean, atmosphere, and ice and bed geometry, i.e., an ice sheet that is in thermal equilibrium with its boundaries. Ice and bed geometry was fixed during spin-up. Letting the ice evolve freely afterward, a marine ice sheet instability starts to unfold, originating from TG. After 100 years, the acceleration of ice loss from the PIG and TG basins was well within the range of present-day observations (Fig. 2) (*1*). We chose this point in time as the starting point of the perturbation through the local addition of ice. The mass deposition took place in a sector covering the coastal region of PIG and TG, which spans the area between −110° and − 97° of longitude and − 76.5° and − 74.5° of latitude (Fig. 1), and was restricted to ice-covered areas that were grounded at the onset of the perturbation. The perturbations were applied for durations of 10, 15, 20, 25, 30, 35, 40, 45, and 50 years, and rates of additional accumulation in that region ranged from 62.5 to 875 Gt year^−1^, yielding a total of 92 simulations. After the end of the perturbation pulse, the original present-day accumulation rates were applied, and the simulations were integrated for 3000 years to investigate the long-term stability of the evolving ice sheet.

Initial bed and ice topography was obtained from the Bedmap2 dataset (*64*). Atmospheric boundary conditions stem from RACMO [version 2.3p2 (*43*)]. RACMO is forced by ERA-Interim reanalysis data at the lateral boundaries, simulating the interaction of the ice sheet with its atmospheric environment, involving relevant processes such as solid precipitation, snow sublimation, and surface meltwater runoff. We averaged the resulting fields of surface mass balance and temperature over the historic period (1979–2016) to obtain a present-day representation of the atmosphere, which was prescribed in the PISM simulations. Oceanic boundary conditions (temperature and sub–ice shelf melt rates) were calculated via PICO (*41*), which, as a module of PISM, simulates the basic vertical overturning circulation in ice shelf cavities. PICO uses a boundary layer melt formulation and extends the box model approach by Olbers and Hellmer (*65*) to two horizontal dimensions, following the shape of the grounding line and the calving front. This way, the model qualitatively reproduces the typical pattern of ice shelf melt, with high melting at the grounding line and low melting or refreezing toward the calving front. PISM supplies the evolving ice shelf geometry to PICO, which, in turn, adjusts in each time step the ocean box geometry to the ice shelf geometry. PICO was forced by circum-Antarctic observations of ocean temperature and salinity (*42*), averaged over the period 1975–2012. The resulting PICO melt rates capture the wide range of observed present-day average melt rates for the individual Antarctic ice shelves (*41*).

## Supplementary Material

http://advances.sciencemag.org/cgi/content/full/5/7/eaaw4132/DC1

Download PDF

## SUPPLEMENTARY MATERIALS

Supplementary material for this article is available at http://advances.sciencemag.org/cgi/content/full/5/7/eaaw4132/DC1 Fig. S1. Observed and modeled ice surface speed. Fig. S2. Cross sections through PIG and TG for a fixed perturbation duration (*T* = 20 years) and a varying rate *R* (increasing from top to bottom), corresponding to the column of black circles in Fig. 4A. Fig. S3. Cross sections through PIG and TG for a fixed perturbation rate (*R* = 250 Gt year^−1^) and a varying duration *T* (increasing from top to bottom), corresponding to the row of black circles in Fig. 4A. Reference (*66*)
