# Supplementary material for: Stabilizing the West Antarctic Ice Sheet by surface mass deposition
Source: Sci Adv. 2019 Jul 17;5(7):eaaw4132. doi: 10.1126/sciadv.aaw4132 (PMC6636986; doi:10.1126/sciadv.aaw4132)
Supplement: http://advances.sciencemag.org/cgi/content/full/5/7/eaaw4132/DC1 [file supp_5_7_eaaw4132__index.html]

Science Advances | Science AdvancesAAASSearchScience AdvancesMenu

## Supplementary Materials

**This PDF file includes:**

- Fig. S1. Observed and modeled ice surface speed.
- Fig. S2. Cross sections through PIG and TG for a fixed perturbation duration (*T* = 20 years) and a varying rate *R* (increasing from top to bottom), corresponding to the column of black circles in Fig. 4A.
- Fig. S3. Cross sections through PIG and TG for a fixed perturbation rate (*R* = 250 Gt yr−1) and a varying duration *T* (increasing from top to bottom), corresponding to the row of black circles in Fig. 4A.
- Reference (*66*)

Download PDF

**Files in this Data Supplement:**

- Adobe PDF - aaw4132\_SM.pdf
