# Supplementary material for: Stabilizing the West Antarctic Ice Sheet by surface mass deposition
Source: Sci Adv. 2019 Jul 17;5(7):eaaw4132. doi: 10.1126/sciadv.aaw4132 (PMC6636986; doi:10.1126/sciadv.aaw4132)
Supplement: Download PDF [file aaw4132_SM.pdf]

Science Advances
AAAS

advances.sciencemag.org/cgi/content/full/5/7/eaaw4132/DC1

Supplementary Materials for

Stabilizing the West Antarctic Ice Sheet by surface mass deposition

Johannes Feldmann, Anders Levermann*, Matthias Mengel

*Corresponding author. Email: anders.levermann@pik-potsdam.de

Published 17 July 2019, *Sci. Adv.* **5**, eaaw4132 (2019)
DOI: 10.1126/sciadv.aaw4132

This PDF file includes:

Fig. S1. Observed and modeled ice surface speed.
Fig. S2. Cross sections through PIG and TG for a fixed perturbation duration (T = 20 years) and a varying rate R (increasing from top to bottom), corresponding to the column of black circles in Fig. 4A.
Fig. S3. Cross sections through PIG and TG for a fixed perturbation rate (R = 250 Gt year^{-1}) and a varying duration T (increasing from top to bottom), corresponding to the row of black circles in Fig. 4A.
Reference (*66*)

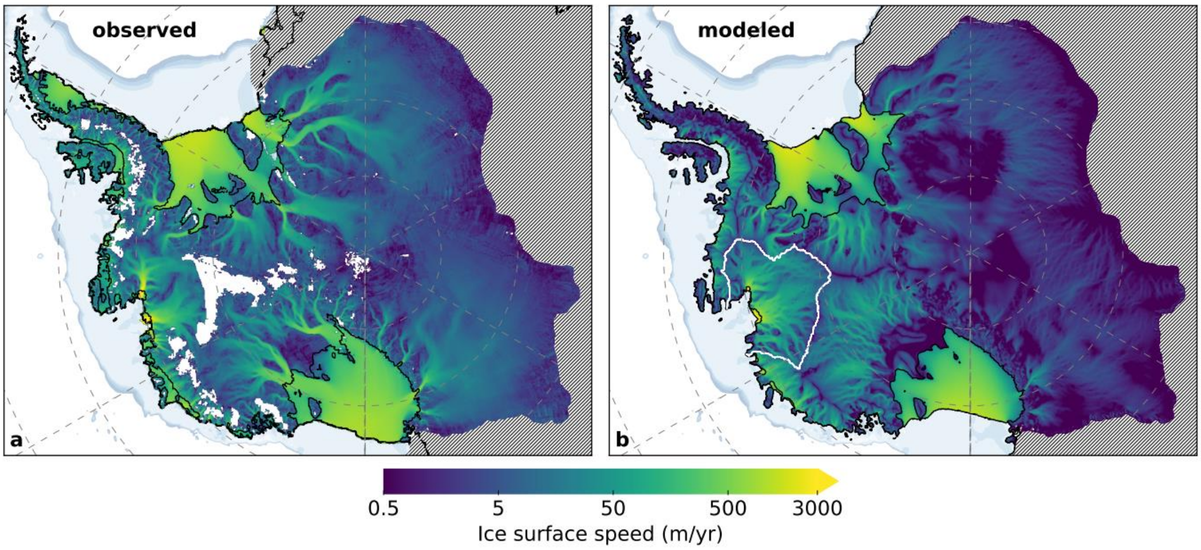

Fig. S1. Observed and modeled ice surface speed. a Observed *(66)* and **b** modeled ice surface speed (colorbar). Continental shelf in blue. The joint catchment basin of PIG and TG is highlighted in white in panel b.

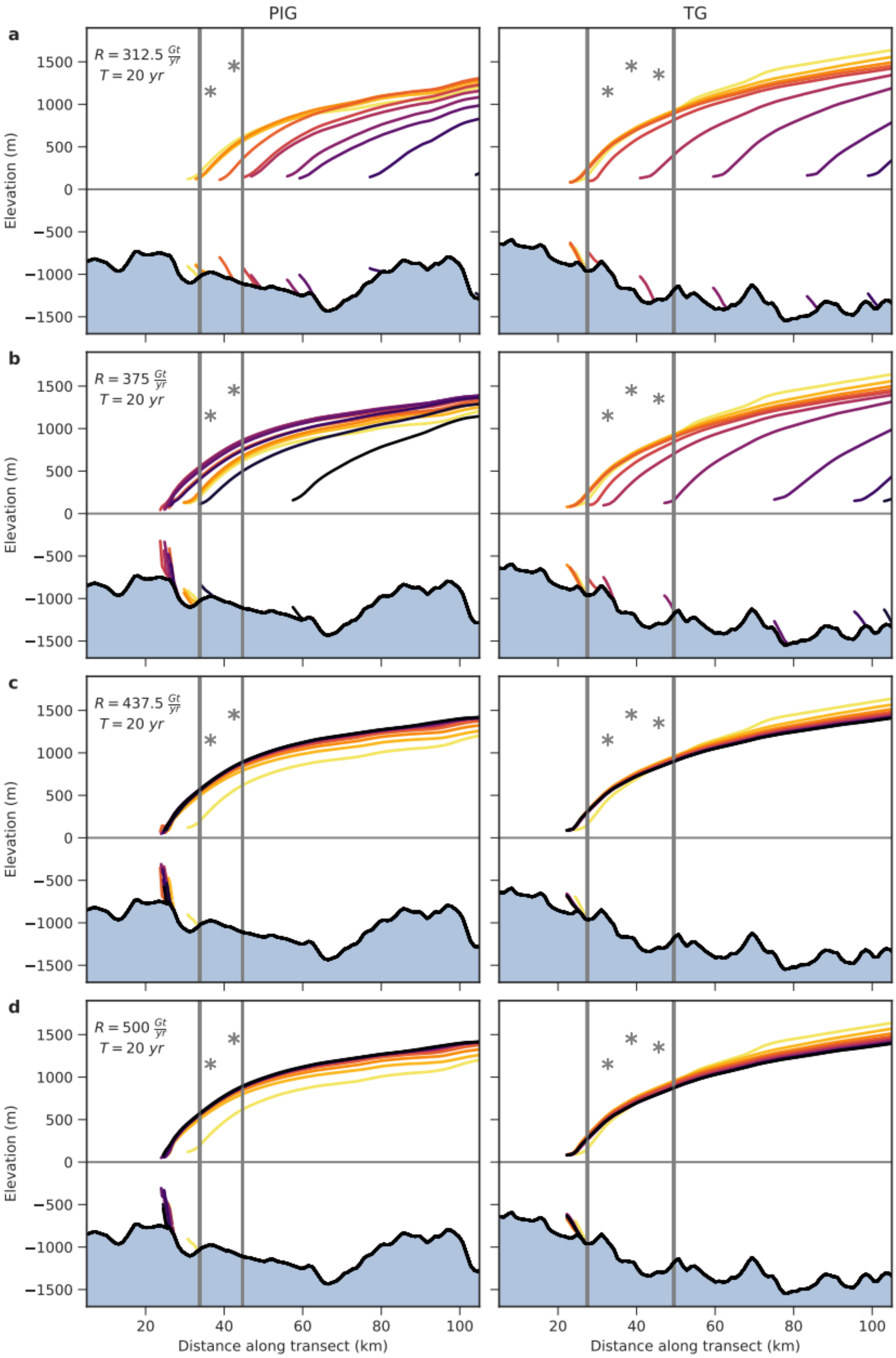

Fig. S2. Cross sections through PIG and TG for a fixed perturbation duration (*T* = 20 years) and a varying rate *R* (increasing from top to bottom), corresponding to the column of black circles in Fig. 4A.

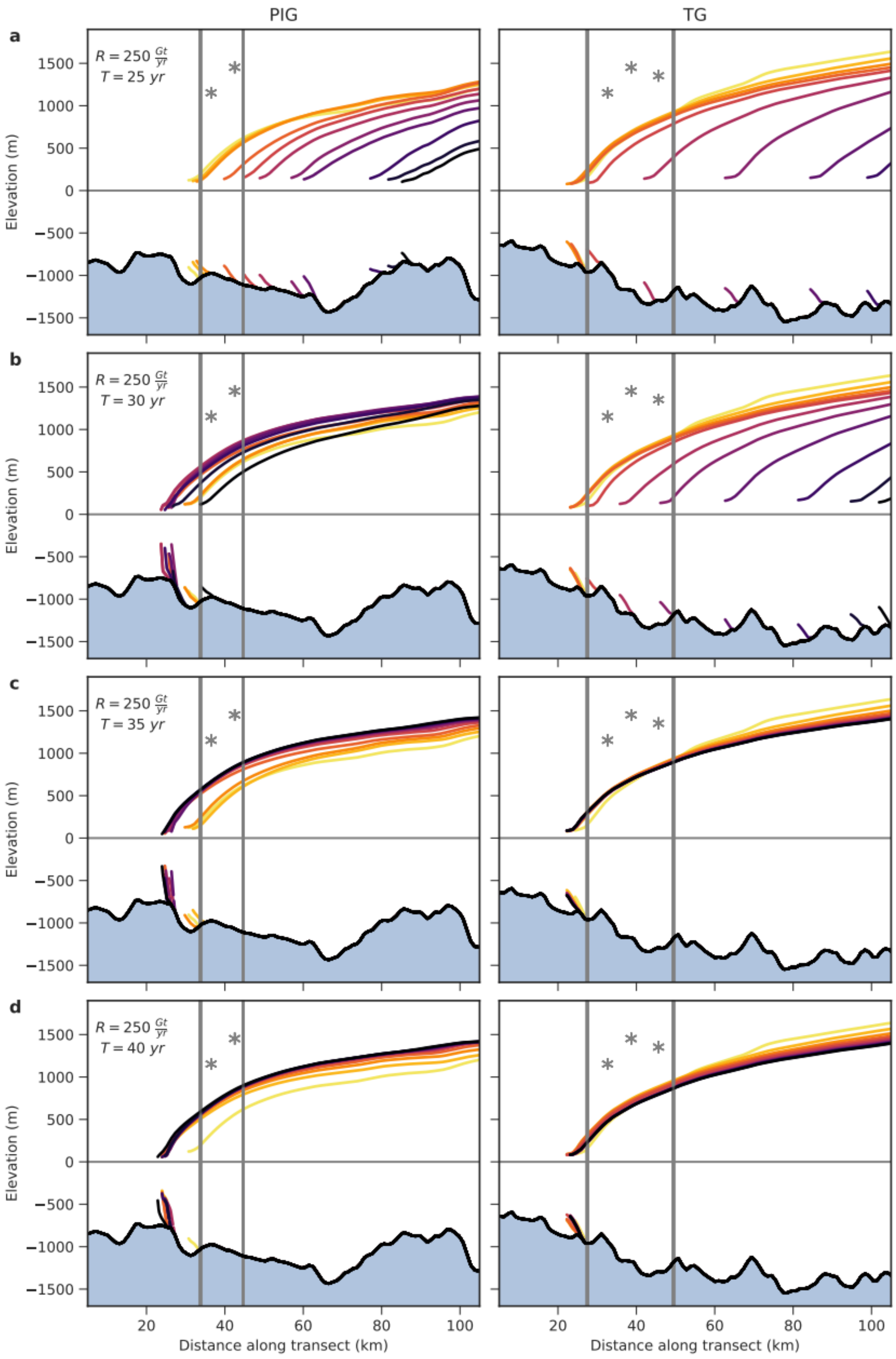

Fig. S3. Cross sections through PIG and TG for a fixed perturbation rate (R = 250 Gt year^{-1}) and a varying duration T (increasing from top to bottom), corresponding to the row of black circles in Fig. 4A.
